# The Optimal Combination of Dietary Starch, Non-Starch Polysaccharides, and Mannan-Oligosaccharide Increases the Growth Performance and Improves Butyrate-Producing Bacteria of Weaned Pigs

**DOI:** 10.3390/ani10101745

**Published:** 2020-09-25

**Authors:** Hua Zhou, Bing Yu, Jun He, Xiangbing Mao, Ping Zheng, Jie Yu, Junqiu Luo, Yuheng Luo, Hui Yan, Daiwen Chen

**Affiliations:** 1 Key Laboratory of Animal Disease-Resistance Nutrition, Chengdu 611130, China; ZHJoseph@outlook.com (H.Z.); ybingtian@163.com (B.Y.); hejun8067@163.com (J.H.); acatmxb2003@163.com (X.M.); zpind05@163.com (P.Z.); yujie@sicau.edu.cn (J.Y.); junqluo2018@tom.com (J.L.); luoluo212@126.com (Y.L.); yan.hui@sicau.edu.cn (H.Y.); 2 Animal Nutrition Institute, Sichuan Agricultural University, Chengdu 611130, China

**Keywords:** starch, non-starch polysaccharides, mannan-oligosaccharide, optimal combination, growth performance, weaned pigs

## Abstract

**Simple Summary:**

Information about the optimal carbohydrate combination for pigs is scarce. This present study explored the effects of different combinations of starch, non-starch polysaccharides, and mannan-oligosaccharide on the growth performance, nutrient digestibility, and microbial communities in weaned pigs, which contributed a novel way of evaluating the carbohydrate quality of the diet for pigs.

**Abstract:**

The present experiment was conducted to dissect the effects of different carbohydrate combinations on the growth performance, nutrient digestibility, and microbial communities in weaned pigs. The combination was optimized by constructing L_9_(3^4^) orthogonal design. Three factors include starch (amylose to amylopectin (AM/AP) ratio 2:1, 1:1, 1:2), non-starch polysaccharides (NSP) (1%, 2%, 3%, a mixture of inulin with cellulose by 1:1), and mannan-oligosaccharide (MOS) (400, 800, 1200 mg/kg) were investigated and nine combinations were implemented under different levels of these factors. One hundred and sixty-two weaned pigs were randomly assigned to nine dietary treatments with six replicates per treatment and three pigs per replicate. Results exhibited that different combinations of starch, NSP, and MOS affected the gain to feed (G:F) (*p* < 0.05), diarrhea incidence (*p* < 0.10), nutrient digestibility (*p* < 0.05), microbial communities, and short-chain fatty acid (SCFA) concentrations (*p* < 0.05). In the present study, taking into account three-way ANOVA, range, and direct analysis, we found that the optimal carbohydrate combination was starch AM/AP 1:1, NSP 3%, MOS 400 mg/kg for weaned pigs. Moreover, feeding this combination diet could promote the growth performance and nutrient digestibility, increase the butyrate-producing bacteria, and to some extent improve lipid metabolism. This study provided a novel way to evaluate the carbohydrate quality in swine production.

## 1. Introduction

Carbohydrates are the most abundant energy sources for pigs, accounting for 60–70% of total energy intake [1]. Notably, the types and structures of carbohydrates are very complicated, and exert different effects on pigs. Previous studies indicated that feeding with starch in different amylose to amylopectin (AM/AP) ratios showed noteworthy physiological responses to growth performance of pigs [2,3]. Emerging evidence reported that starch in higher AM/AP ratio increased the average daily gain and feed efficiency of finishing pigs [4,5]. Moreover, starch in higher AM/AP ratio could improve intestinal health by regulating gut microbiota, promoting intestinal morphology, and increasing intestinal functions related gene expressions [6,7]. Similarly, the increase of dietary amylose caused the reduction of conditioned pathogens and induced the increase of some beneficial bacteria [8]. Dietary fibers, mainly made up of non-starch polysaccharides (NSP), have been demonstrated in promoting gastrointestinal health both in human and animals [9,10]. Intake of the right amount of dietary fiber did not affect the growth performance of piglets [11]. Moreover, accumulating evidence showed that dietary fiber improved the intestinal morphology and barrier function, decreased diarrhea incidence, and enhanced the proportion of *Lactobacillus* in gastrointestinal tract of piglets [12,13,14]. In addition, feeding with both soluble and insoluble fiber was more effective than supplementation alone to improve nutrient absorption and intestinal barrier function of pigs [15]. Additionally, dietary fiber-deprived microbiota increased the susceptibility of pathogens and impaired the barrier function of colonic mucosa [16]. Furthermore, some oligosaccharides and polysaccharides are recognized as prebiotics for human and animals, which could be used to stimulate the proportion of *Lactobacillus* and *Bifidobacterium* [17,18,19]. Mannan-oligosaccharide (MOS) is derived from yeast cell wall, and could improve the balance of gut microflora and improve intestinal immune function [20]. Importantly, dietary supplement with MOS has been shown to increase the growth performance of pigs [21,22,23,24]. Taken together, starch, dietary fiber, and oligosaccharides have been well documented and exhibited beneficial role on pig’s growth and intestinal health. However, until now, information about the optimal combination of starch, dietary fiber, and oligosaccharide for pigs is limited.

Therefore, the purpose of present study was to fill in the gap of knowledge about the effects of different combinations of starch, dietary fiber, and oligosaccharide on the growth performance, nutrient digestibility, short-chain fatty acid (SCFA) production, and microbial community of weaned pigs, and to evaluate the optimal carbohydrates combination for weaned pigs. An optimal combination of starch, dietary fiber, and oligosaccharide may be a novel metric for assessing carbohydrate quality in relation to swine production.

## 2. Materials and Methods

Experimental proposals and procedures used in the present experiment were conducted in accordance with the Animal Care and Use Committee of Sichuan Agricultural University (Ya’an, China) under permit number DKY-B20131704.

### 2.1. Experimental Design, Animals, and Diet

An orthogonal experiment consisting of L_9_(3^4^) orthogonal array was used in this study. The design involved three factors, and each factor has three levels. Namely, starch (AM/AP ratio 2:1, 1:1, 1:2), NSP levels (1%, 2%, 3%, a mixture of inulin with cellulose by 1:1), and MOS contents (400, 800, 1200 mg/kg). Regardless of the interactions between the three factors, the orthogonal array table L_9_(3^4^) was used as experimental design and listed in Table 1. In total, 162 crossbred Duroc × (Landrace × Yorkshire) pigs weaned at 21 ± 1 d were provided by a commercial farm and housed in a temperature and light-controlled nursery house with completely slotted floors, and fed a common pre-starter diet for 5-days adaptation. Pigs with an initial average body weight (BW) of 6.70 ± 0.34 kg were randomly assigned to one of nine dietary treatments with six replicate pens (three pens were barrows and the other three pens were gilts) per treatment and three pigs per pen. Each pen (1.8 × 2.5 m) was equipped with a single nipple waterer and a one-sided self-feeder (Laien, Animal Husbandry Engineering Co. LTD., Chengdu, China). Pigs were allowed ad libitum access to water and feed. This experiment lasted 28 d. Room temperature initially was set at 28 °C for the first week and gradually reduced to 25 °C by the end of the experiment. Meal diets were formulated to meet or exceed the nutrient recommendation of NRC (2012) for pigs weighing 10–20 kg. The MOS was added to experimental diets by substitution of maize. The contents of crude protein and net energy were equalized by adjusting the proportion of maize and soybean meal in each diet. The compositions of ingredients, and calculated and analyzed nutrients of diets, are presented in Table 2 and Table 3. No medicines or antibiotics were used. The purity of amylose, amylopectin, inulin (the degree of polymerization of 2–60, and an average of 12), cellulose, and MOS was 68.5%, 95%, 85%, 99%, 95%, respectively, and were obtained from Kang Biological Products Co., Ltd. (Chicago, IL, USA), Fuyang Biotechnology Co., Ltd. (Dezhou, China), VILOF Group Co., Ltd. (Beijing, China), Tianli Medical Supplements Co., Ltd. (Qufu, China), Alltech Biological Products Co., Ltd. (Beijing, China), respectively.

### 2.2. Sampling and Measurements

Before the experiment began, the experimental diets were sampled once and stored at −20 °C for chemical analysis. Feces were collected on d 24–28 from each pen, added with 10% hydrochloric acid to fix excreta nitrogen after collection, and were dried in a forced-air oven (65 °C) for 72 h. Samples of feed and feces were ground through a 1-mm screen until analyzed for dry matter (DM), crude protein (CP), ether extract (EE), gross energy (GE), crude ash (Ash), and crude fiber (CF). On the 28th day of experiment, one pig with average BW of each pen was selected to obtain blood samples via anterior vena cava before weighing (12 h after feeding). Blood was collected into vacuum tubes without anticoagulant (Kangjian Medical Apparatus Co., Ltd., Taizhou, China), and centrifuged at 3000× *g* for 15 min to obtain serum, and stored at −80 °C for further analysis. Then the selected pigs were euthanized by intravenous injection of sodium pentobarbital (200 mg/kg BW) according to Chen et al. (2013) [25]. The abdomen was opened to remove the small intestinal segment immediately, the samples from middle sections (4 cm) of duodenum, jejunum, and ileum were collected and stored in 4% paraformaldehyde solution for microscopic measurement of intestinal morphology. This was followed by collecting approximately 4 g digesta from caecum and proximal colon, respectively, kept in sterile tubes and immediately frozen at −80 °C until analysis of SCFA concentrations. Meanwhile, the digesta samples in proximal colon of selected pigs were collected for bacterial community measurement.

### 2.3. Growth Performance

The pigs were weighed individually on d 0 and 28 of the experiment and feed intake per pen was measured daily to determine average daily feed intake (ADFI), average daily weight gain (ADG), and the ratio of gain to feed (G:F). Pigs showing liquid feces or loose feces were evaluated as having diarrhea, and the diarrhea of each pen were observed by two observers blinded to treatments at the same time of every morning, noon, and evening for 28 days to determine the number of pigs with diarrhea. The diarrhea incidence was calculated as follows: diarrhea incidence (%) = A/(B × 28 d) × 100, where A = total number of pigs per pen with diarrhea, and B = number of pigs per pen, which was suggested by Zhang et al. (2015) [26].

### 2.4. Digestibility Determination

The apparent total tract digestibility (ATTD) was determined using acid insoluble ash (AIA) as a digestibility indicator. AIA in feed and feces were measured by the method of Chinese National Standard (GB/T 23742). Chemical analysis of feed and fecal samples was detected as follows. DM (method 930.15), Ash (method 942.05), EE (method 945.16), CP (method 990.03), and CF (method 920.98) were assessed according to the procedures of AOAC (1995) [27]. The GE of feed and fecal samples were determined using an adiabatic oxygen bomb calorimetry (Parr Instrument Co., Moline IL). The digestibility was calculated by the following formula: ATTD (%) = (100 − A1/A2 × F2/F1 × 100), in which A1 represents the AIA content of the feed; A2 represents the AIA content of the feces; F1 represents the nutrient content of the feed; F2 represents the nutrient content of the feces.

### 2.5. Serum Sample Analysis

The contents of blood glucose (Glu), cholesterol (TC), triglycerides (TG), high density lipoprotein-cholesterol (HDL-c), low density lipoprotein-cholesterol (LDL-c) were detected by the commercial kits (Jiancheng Bioengineering Institute, Nanjing, China) with UV-VIS Spectrophotometer (UV1100, MAPADA, Shanghai, China) according to the manufacturer’s instructions. Each parameter was determined in triplicate simultaneously on the same plate. Additionally, the differences among parallels must be small (coefficient of variation was less than 10%) to guarantee the reproducibility of repeated measurements.

### 2.6. Histological Analysis

The morphology measurements of the villus height (VH) and crypt depth (CD) were conducted according to Touchette et al. (2002) [28]. Briefly, 4-cm of duodenum, jejunum, and ileum were washed with cold sterile saline and fixed with 4% paraformaldehyde solution, and then were dehydrated and embedded in paraffin wax before transverse sections were cut. The preserved samples were stained with hematoxylin and eosin. Twelve well-orientated sections of height villi and their adjoint crypts in each sample were measured with Image Pro Plus software (Version 6.0, Media Cybernetics, Bethesda, MD, USA) at 40× magnification.

### 2.7. Determination of Bacterial Community and Data Analysis

Total DNA of each digesta sample was isolated using QIAamp stool DNA Mini kit (QIAGEN, Valencia, CA, USA). The concentration and purity of genomic DNA were detected by NanoDrop ND-2000 Spectrophotometer (NanoDrop, Hilden, Germany). The integrity of genomic DNA was measured using electrophoresis on 1% agarose gels. Sequencing was determined by the Novogene Bioinformatics Technology Co. Ltd. (Beijing, China). DNA library was generated before high-throughput sequencing as previously reported [29]. The library was sequenced on the Illumina HiSeq platform and using 250-bp paired-end reads strategy. The resulting sequences were clustered into operational taxonomic units (OTUs) using USEARCH drive at 97% sequence similarity, and a representative sequence was selected. The relative abundance of each OTU was examined at different taxonomic levels. Species annotation of OTU representative sequences was identified with RDP Classifier method and Green Gene database. The populations of bacterial community in colonic digesta of weaned pigs at the phyla, class, order, family, and genera levels were analyzed by the method of Kruskal–Wallis. The abundances of bacteria at the phylum, class, order, and family levels were shown as bar plots. Log10-transformation was used on the genus relative abundance data matrix for the heatmaps representation, which allowed visualizing differences or similarities between treatments. The Beta diversity of nonmetric multidimensional scaling (NMDS) was analyzed, and the plots were produced by Bray–Curtis distances. The plots were visualized using R software (Package ape).

### 2.8. Analysis of SCFA Concentrations

The sample pretreatment of serum, and cecal and colonic digesta was according to the previous method [30]. The concentrations of SCFAs (acetate, propionate, and butyrate) in serum and digesta of cecum and colon were determined by the gas chromatography system (CP-3800 GC, Varian, Inc., Walnut Creek, CA, USA), and following the instructions described by Franklin et al. (2002) [31].

### 2.9. Statistical Analysis

The GLM models of SAS 9.2 (SAS Institute, Inc., Cary, NC, USA) and Tukey’s tests were used to analyze these experimental data. For data on growth performance and nutrient digestibility, the pen was used as the experimental unit. For other indexes (serum parameters, intestinal morphology, and SCFA concentrations), each pig was regarded as the statistical unit. Moreover, range and three-way ANOVA analyses were performed on growth performance data. The results were presented as means and standard error of the mean (SEM). Statistical significance was considered as *p* < 0.05 and was considered a trend at 0.05 ≤ *p* < 0.10.

## 3. Results

### 3.1. Growth Performance

As shown in Table 4, feeding with different combinations of starch, NSP, and MOS significantly affected the G:F of weaned pigs (*p* < 0.05), and tended to influence the diarrhea incidence (*p* < 0.10). Distinctly, we could observe the final BW, ADFI, and ADG in the T6 group (AM/AP 1:1, NSP 3%, and MOS 400 mg/kg) were the highest among the treatments. Likewise, the G:F was higher and diarrhea incidence was lower in the T6 group compared to other groups except T3 (AM/AP 2:1, NSP 3%, and MOS 1200 mg/kg). Meanwhile, taking pigs’ ADFI, G:F, and diarrhea incidence as indicators, range analysis indicated that starch in different AM/AP has the greatest effect on them, and followed by NSP levels and MOS contents. Besides, taking pigs’ final BW, and ADG as indicators, range analysis demonstrated that the NSP level has the greatest effect on them, and followed by starch in different AM/AP and MOS contents (Table 5). In addition, taking final BW, ADG, G:F, and diarrhea incidence into range analysis, the diet of starch AM/AP 2:1, NSP 3%, and MOS 400 mg/kg was evaluated as the best combination, while taking ADFI into range analysis the diet of starch AM/AP 1:1, NSP 3%, and MOS 1200 mg/kg was assessed as the best combination. Moreover, through three-way ANOVA analysis, we found that starch in different AM/AP tended to influence the final BW and ADG (*p* < 0.10), and has a marked effect on G:F and diarrhea incidence (*p* < 0.05). Additionally, the levels of NSP tended to influence ADG, G:F, and diarrhea incidence (*p* < 0.10). Besides, the contents of MOS tended to influence G:F (*p* < 0.10) (Table 6).

### 3.2. Apparent Total Tract Digestibility

The nutrient digestibility is presented in Table 7, the digestibility of DM, EE, Ash, GE, and CP were apparently affected by different combinations of starch, NSP, and MOS (*p* < 0.05). Meanwhile, the digestibility of DM, EE, Ash, and GE in the T6 group were increased (*p* < 0.05) compared to T7, T8, and T9 groups. Besides, the digestibility of DM, EE, Ash, GE, and CP in the T6 group were higher than (*p* < 0.05) T1, T2, and T3 groups. Moreover, the digestibility of all nutrients in the T6 group was the greatest among the treatments.

### 3.3. Serum Biochemical Parameters

The content of LDL-c was markedly affected by different combinations of starch, NSP, and MOS (*p* < 0.05) (Table 8). Notably, the concentration of LDL-c in the T9 group was the lowest among the treatments. However, no difference was observed in the concentration of LDL between T6 and T9 (*p* > 0.05), and the concentration of LDL-c in the T6 group was lower than other treatments.

### 3.4. Intestinal Morphology

As presented in Table 9, there was an influence of different combinations of starch, NSP, and MOS on the villi height in duodenum (*p* < 0.10). Additionally, we observed the villi height and VH:CD in duodenum of T2 and T3 groups were higher than other treatments. Moreover, the villi height in duodenum of the T6 group was generally greater than other groups except T2 and T3.

### 3.5. SCFA Contents in Serum and Hindgut

According to Table 10, feeding with different combinations of starch, NSP, and MOS impacted the concentrations of butyrate in serum and acetate in digesta of cecum and colon (*p* < 0.05). Meanwhile, the content of serum butyrate in the T6 group was the greatest among the treatments, and the concentration of serum butyrate in the T6 group was markedly increased (*p* < 0.05) compared with T7, T8, and T9. Moreover, the concentrations of acetate and propionate in cecal and colonic digesta of the T6 group were the lowest among the treatments.

### 3.6. 16S rRNA Analysis of Bacterial Communities.

**Figure 1:**
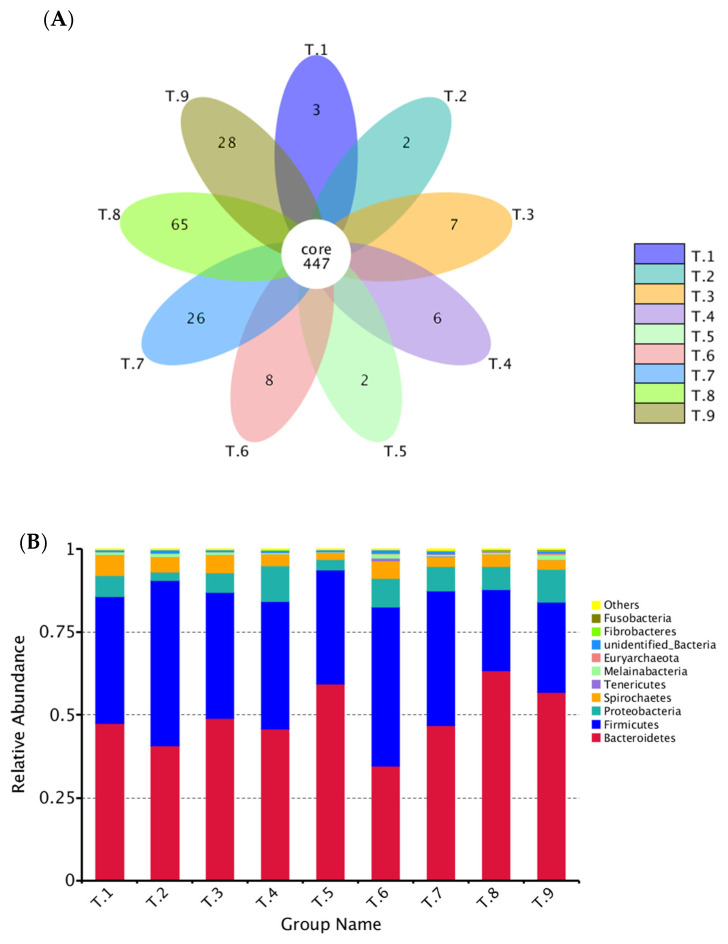
The bacterial OTU community composition and microbial diversity at the phylum level in colonic digesta of weaned piglets. (**A**) Flower diagrams for bacterial OTU in nine dietary treatments. (**B**) Microbial community bar-plot of the top 10 at the phylum level.

**Figure 2:**
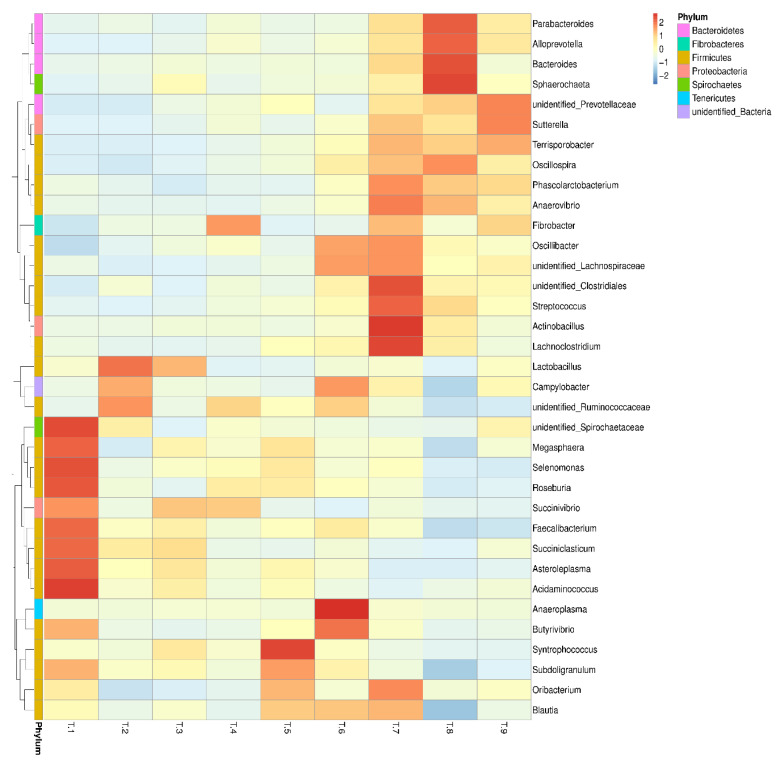
The bacterial community heatmap of the top 35 bacteria at genus level. The different colors represented the relative abundance of bacteria in nine dietary treatments.

**Figure 3:**
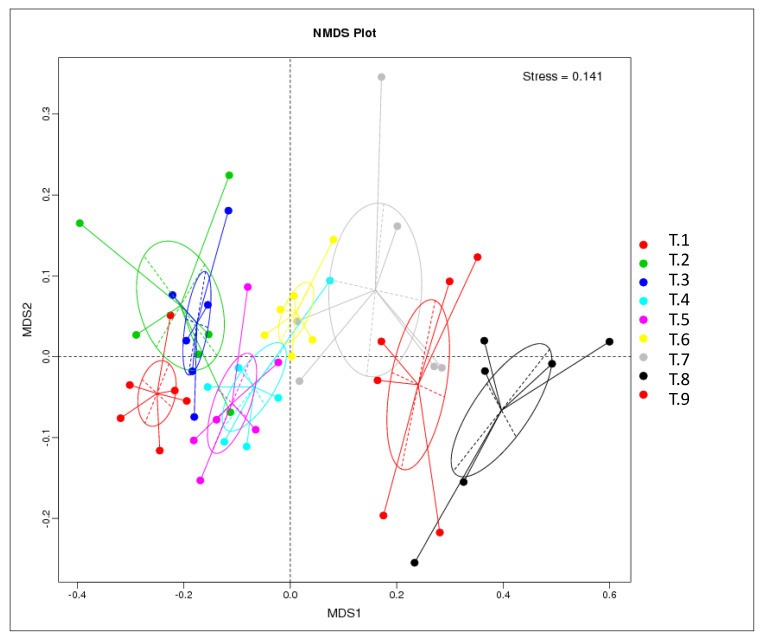
Nonmetric multidimensional scaling analysis to visualize the Bray–Curtis distances of colonic digesta microflora in T1–T9 groups.

The colonic digesta samples of pigs in T1–T9 groups showed 450, 449, 454, 453, 449, 455, 473, 512, and 475 OTUs, respectively. Among them, 447 shared common bacteria and total 147 individual bacteria were isolated in nine dietary treatments (Figure 1A). At the phylum level, all of the groups were dominated by *Firmicutes*, *Bacteroidetes*, *Proteobacteria*, *Spirochaetes*, and *Tenericutes* (Figure 1B). The numerical composition of bacterial community on the phylum level was analyzed (Supplementary Table S1), and the abundances of *Firmicutes* and *Bacteroidetes* were markedly affected by different combinations of starch, NSP, and MOS (*p* < 0.05). Meanwhile, the abundances of *Firmicutes* and *Bacteroidetes* in the T6 group were the greatest and lowest among the groups, respectively. Similarly, at the class, order, and family levels, feeding with different combinations of starch, NSP, and MOS obviously impacted the abundances of *Bacteroidia*, *Bacteroidales*, and *Prevotellaceae* (*Bacteroidetes*) (*p* < 0.05), and all of them in the T6 group were lower than those in other groups. Likewise, the abundances of *Clostridia*, *Clostridiales*, and *Ruminococcaceae* (*Firmicutes*) were apparently affected by different combinations of starch, NSP, and MOS (*p* < 0.05), and all of them in the T6 group showed a higher abundance in comparation with other groups (Figures S1–S3; Tables S2–S4). At the genera level, the abundances of top 35 bacterial communities were presented in the heatmap (Figure 2). The numerical composition of top 15 bacterial community on the genera level was analyzed (Table S5). Feeding with different combinations of starch, NSP, and MOS significantly affected the abundances of *Prevotella_9*, *Ruminococcus_2*, *Alloprevotella*, *Prevotellaceae_NK3B31_group*, *Prevotella_2*, *Lactobacillus Prevotella_1*, *Succiniclasticum* (*p* < 0.05), and the abundances of *Prevotella_9* (*Bacteroidetes*) and *Ruminococcus_2* (*Firmicutes*) in the T6 group was relatively lower and higher than that in other groups, respectively. Furthermore, the NMDS analysis was carried out to determine the extent of the difference between microbial communities (Figure 3). The gut microbial composition from T1 to T9 group could be divided into nine different clusters and could be separated generally by NMDS analysis (stress = 0.141).

## 4. Discussion

Polymeric carbohydrates, starch, and NSP represent the largest proportion of diets and are the major energy contributor for pigs [32]. Notably, previous investigation showed that carbohydrate quality rather than quantity was more important in preventing type 2 diabetes [33]. Moreover, it has been established that diets with high starch, low fiber, and a high ratio of starch to cereal fiber were associated with a higher risk of type 2 diabetes [34]. Of note, in traditional swine nutrition, analyses of starch and fiber have focused on measuring of its quantity, while the information about the best quality carbohydrates for pigs are poorly understood. As we know, starch and NSP are the main energy source for pigs, and the undigested carbohydrates are the fermentative substrates of hindgut microbes. Meanwhile, MOS was considered as prebiotic and widely used in the swine industry. Therefore, the present study was conducted to dissect the effects of different combinations of starch, NSP, and MOS on the growth performance, nutrient digestibility, SCFA production, and microbial community, and to establish an optimal carbohydrates combination for weaned pigs. The orthogonal array design is a scientific method of reducing experimental treatments, enhancing efficiency, and optimizing design projects. In the present work, the orthogonal array design was used to explore the effects of different combinations of starch, NSP, and MOS on weaned pigs. Noteworthy, through three-way analysis, we obtained that starch in different AM/AP, NSP levels, and MOS contents both have marked effects on or tended to influence G:F, which implied that G:F was the most important indicator for evaluating the optimal carbohydrates combination for weaned pigs. Intriguingly, taking G:F into consideration, by range analysis, we found the diet of starch AM/AP 2:1, NSP 3%, and MOS 400 mg/kg was the best combination, while not included in the experimental treatments. Hence, further research on the optimal carbohydrate combinations for weaned pigs are clearly warranted. Of note, close to the best combination is the T3 diet (starch AM/AP 2:1, NSP 3%, and MOS 1200 mg/kg). However, taking into account direct analysis, although we found that the T3 group had the greatest G:F and lowest diarrhea incidence, it was similar to the T6 group. Moreover, the final BW, ADFI, and ADG in the T6 group were the highest among the treatments. Furthermore, as the price of high AM maize starch is higher than high AP and conventional maize starch, formulating diets with greater content of AM will increase the cost in swine production. Collectively, based on the growth performance and the cost, we evaluated that T6 diet (starch AM/AP 1:1, NSP 3%, and MOS 400 mg/kg) was the optimal carbohydrate combination for weaned pigs.

The nature of different types of starch is the difference in AM/AP. As the molecular configuration and structure are different, starch with high amylopectin is easily digested in the foregut, while amylose is the opposite [35]. Thus, the digestibility and physiological functions of dietary starch mainly depended on the ratio of AM/AP [36]. In the current study, the AM/AP of starch in T6 diet is 1:1, higher than the ratio of 1:2 in T7, T8, and T9 groups. Noteworthy, we found the final BW, ADFI, and ADG of the T6 group were greater than those in other groups. Similarly, previous work indicated that the ADG of finishing pigs tended to enhance as the ratio of AM/AP increased [2]. Likewise, recent evidence revealed that the ADG of finishing pigs tended to enhance, accompanied by AM/AP (1:1, 1:2, 1:3, 1:4) increase [5]. Nevertheless, the growth performance of T1 and T2 groups (with 2:1 ratio of AM/AP) were not higher than the T6 group. Notably, the health benefits of dietary fibers have been well discussed, as fiber-dependent microbiota affect the barrier function of colonic mucosa and the susceptibility to pathogens [16]. In addition, dietary fiber was a critical ingredient in the diet which promoted the intestinal development and health of pigs [12,13,14]. Consequently, the content of NSP in T1 and T2 diets was lower than the T6 diet, which might be the reason for the growth performance being lower than the T6 group. The MOS was the most extensively investigated candidate for alternatives to antibiotics [37]. It has been reported that MOS supplementation provided benefits in swine production, and was similar to that of antimicrobial growth enhancers [38,39]. Yet, the content of NSP in the T3 diet was the same, and the levels of MOS in the T3 diet was higher than the T6 diet, while no difference was observed on growth performance between them. A previous report in our laboratory observed that piglets fed the high AM diet had significantly decreased nutrient digestibility [40]. Thus, the reason may be that the starch AM/AP in T3 was greater than T6, and too much amylose impaired the nutrient digestibility. Indeed, in the current study, the nutrient digestibility of the T3 group was markedly decreased compared to the T6 group. In addition, in the present study, the levels of MOS also tended to influence the G:F of weaned pigs, and the 400 mg/kg was observed as the optimal level. Economically, the addition level of MOS in 400 mg/kg has lower cost compared with the other two levels. Importantly, the intestines play a vital role in the digestion and absorption, as based on the villous and crypt, which exerts the function to expand the exchange of the mucosal surface area in preparation for nutrient load [41]. Moreover, increasing the villus height and decreasing crypt depth in intestine suggested an enhancement of surface area capable of greater absorption of available nutrients [42]. In our study, T2 and T3 groups had higher villous height and VH:CD in duodenum and jejunum than the T6 group. Conversely, we measured the nutrient digestibility in the T6 group, and found it was the greatest among the treatments and apparently higher than T2 and T3 groups. Herein, further well controlled studies are urgently needed.

The gut microbiota provides essential capacities for the fermentation of undigestible carbohydrates into SCFAs [43]. An increase butyrate was regarded beneficial to the host by stimulating the cell proliferation and differentiation, and improving the colonic barrier functions [44,45]. Indeed, butyrate could promote the biosynthesis of defense peptides to improve the immunity of hosts and prevent infectious diseases [46]. Notably, the T6 group had the greatest content of serum butyrate among the treatments. Increased the degradation of fiber components might be the cause for the result of increase of butyrate in serum. The *Rumininococcaceae* was considered as fibrolytic bacteria to ferment the complex components of plant cell walls. Noteworthy, our results showed the abundance of *Rumininococcaceae* in the T6 group was the greatest among the treatments. In addition, the present study observed the abundances of *Firmicutes* and *Bacteroidetes* represented approximately 85% of total gene sequences in colonic digesta of weaned pigs, in agreement with previous research [47]. Meanwhile, the gut microbial composition from T1 to T9 groups could be separated generally by NMDS analysis, and the abundance of *Firmicutes* in the T6 group was the greatest among the treatments. Of note, *Firmicutes* was the predominant butyrate-producing bacteria [44,48]. Taken together, the enhanced concentration of butyrate in serum of the T6 group was possibly due to increases in the abundances of butyrate-producing bacteria and fibrolytic bacteria. Additionally, propionate and acetate were taken up by the liver and used as substrates for gluconeogenesis and lipogenesis [49]. Remarkably, we detected that the concentrations of acetate and propionate in cecum and colon of the T6 group were the lowest among the treatments. Consistently, the abundance of *Bacteroidetes* in the T6 group was lower than other groups, as propionate was mainly produced by *Bacteroidetes* [44,48]. Furthermore, LDL-c can transport cholesterol lipids outside the liver for lipid synthesis, increasing lipid deposition. In the present study, the concentration of LDL-c in the T6 group was similar with T9, while lower than other groups. Collectively, these demonstrated that the T6 diet could promote the abundance of butyrate-producing bacteria and to some extent improve lipid metabolism of weaned pigs.

## 5. Conclusions

In the present study, we identified that the growth performance, nutrient digestibility, microbial community, and SCFA concentrations of weaned pigs were significantly affected by different combinations of starch, NSP, and MOS. Moreover, when the dietary carbohydrates combination was the T6 diet, it could promote the growth performance and nutrient digestibility, increase the abundances of butyrate-producing bacteria, and to some extent improve lipid metabolism of weaned pigs. In summary, we found that the T6 diet (starch AM/AP 1:1, NSP 3% (mixture of inulin with cellulose by 1:1), MOS 400 mg/kg) was the optimal carbohydrates combination for weaned piglets. Importantly, these findings provided a novel way to evaluate the carbohydrate quality of the entire diet for pigs.

## Figures and Tables

**Table 1:** L_9_(3^4^) orthogonal array experimental parameter assignment.

Groups	A	B	C
	Different Ratio of Amylose to Amylopectin	Levels of NSP (Mixture of Inulin with Cellulose by 1:1)	Levels of MOS
T1	2:1 (A1)	1% (B1)	400 mg/kg (C1)
T2	2:1 (A1)	2% (B2)	800 mg/kg (C2)
T3	2:1 (A1)	3% (B3)	1200 mg/kg (C3)
T4	1:1 (A2)	1% (B1)	800 mg/kg (C2)
T5	1:1 (A2)	2% (B2)	1200 mg/kg (C3)
T6	1:1 (A2)	3% (B3)	400 mg/kg (C1)
T7	1:2 (A3)	1% (B1)	1200 mg/kg (C3)
T8	1:2 (A3)	2% (B2)	400 mg/kg (C1)
T9	1:2 (A3)	3% (B3)	800 mg/kg (C2)

NSP: non-starch polysaccharides; MOS: mannan-oligosaccharide.

**Table 2:** Ingredients composition of experimental diets (as-fed basis).

Ingredients, %	Different Combinations of Starch, NSP, and MOS								
	T1	T2	T3	T4	T5	T6	T7	T8	T9
Maize	16.71	15.82	14.93	17.62	15.68	14.06	17.11	15.33	13.47
Puffed maize	10.00	10.00	10.00	10.00	10.00	10.00	10.00	10.00	10.00
Soybean meal	13.20	13.35	13.50	13.05	13.36	13.65	13.12	13.43	13.74
Puffing of soybean	7.00	7.00	7.00	7.00	7.00	7.00	7.00	7.00	7.00
Soy protein concentrate	5.00	5.00	5.00	5.00	5.00	5.00	5.00	5.00	5.00
Whey powder	5.00	5.00	5.00	5.00	5.00	5.00	5.00	5.00	5.00
Fish meal	3.00	3.00	3.00	3.00	3.00	3.00	3.00	3.00	3.00
Plasma protein powder	3.00	3.00	3.00	3.00	3.00	3.00	3.00	3.00	3.00
Glucose	2.00	2.00	2.00	2.00	2.00	2.00	2.00	2.00	2.00
Soybean oil	0.20	0.85	1.50	0.30	0.84	1.40	0.25	0.80	1.35
Conventional maize starch				11.90	12.35	12.60			
High amylose maize starch	30.70	29.75	28.80	17.90	17.50	17.10	3.80	3.80	3.78
High amylopectin maize starch							26.45	26.45	26.45
Cellulose	0.50	1.00	1.50	0.50	1.00	1.50	0.50	1.00	1.50
Inulin	0.50	1.00	1.50	0.50	1.00	1.50	0.50	1.00	1.50
Mannan-oligosaccharide	0.04	0.08	0.12	0.08	0.12	0.04	0.12	0.04	0.08
Limestone	0.45	0.45	0.45	0.45	0.45	0.45	0.45	0.45	0.45
Dicalcium phosphate	1.25	1.25	1.25	1.25	1.25	1.25	1.25	1.25	1.25
L-Lysine-HCl	0.29	0.29	0.29	0.29	0.29	0.29	0.29	0.29	0.29
DL-Methionine	0.12	0.12	0.12	0.12	0.12	0.12	0.12	0.12	0.12
L-Threonine	0.13	0.13	0.13	0.13	0.13	0.13	0.13	0.13	0.13
L-Tryptophan	0.01	0.01	0.01	0.01	0.01	0.01	0.01	0.01	0.01
Vitamin premix ^‡^	0.05	0.05	0.05	0.05	0.05	0.05	0.05	0.05	0.05
Mineral premix ^†^	0.45	0.45	0.45	0.45	0.45	0.45	0.45	0.45	0.45
NaCl	0.25	0.25	0.25	0.25	0.25	0.25	0.25	0.25	0.25
Choline chloride	0.15	0.15	0.15	0.15	0.15	0.15	0.15	0.15	0.15
Total	100	100	100	100	100	100	100	100	100

NSP: non-starch polysaccharides; MOS: mannan-oligosaccharides. ^‡^ Provided the following per kilogram of diet: vitamin A, 8000 IU; vitamin D_3_ 2000 IU; vitamin E, 20 IU; vitamin B_1_, 1.5 mg; vitamin B_2_, 5.6 mg; vitamin B_12_, 0.02 mg; vitamin B_6_, 1.5 mg; vitamin K3, 2 mg; calcium pantotenate, 10 mg; nicotinic acid, 15 mg; biotin, 0.1 mg; folic acid, 0.6 mg. ^†^ Supplemented the following per kilogram of diet: Fe, 100 mg as FeSO_4_; Cu, 20 mg as CuSO_4_.5H_2_O, Zn, 100 mg as ZnSO_4_, Mn, 60 mg as MnSO_4_, I, 0.3 mg as KI, and Se, 0.3 mg as Na_2_SeO_3_.

**Table 3:** Calculated and analyzed nutritional content of experimental diets (as-fed basis).

Nutrient Concentrations	Different Combinations of Starch, NSP, and MOS								
	T1	T2	T3	T4	T5	T6	T7	T8	T9
Calculated values ^‡^									
CP, %	19.00	19.00	19.00	19.00	19.00	19.00	19.00	19.00	19.00
NE, MJ/kg	11.07	11.07	11.07	11.07	11.07	11.07	11.07	11.07	11.07
CF, %	2.30	3.22	4.13	2.31	3.21	4.12	2.31	3.22	4.12
SID-Lysine, %	1.23	1.23	1.23	1.23	1.23	1.23	1.23	1.23	1.23
SID-Methionine, %	0.36	0.36	0.36	0.36	0.36	0.36	0.36	0.36	0.36
SID-Threonine, %	0.73	0.73	0.73	0.73	0.73	0.73	0.73	0.73	0.73
SID-Tryptophan, %	0.20	0.20	0.20	0.20	0.20	0.20	0.20	0.20	0.20
Calcium, %	0.70	0.70	0.70	0.70	0.70	0.70	0.70	0.70	0.70
Available P, %	0.45	0.45	0.45	0.45	0.45	0.45	0.45	0.45	0.45
Analyzed values									
CP, %	20.46	20.71	20.43	20.39	20.45	20.23	20.39	20.57	20.23
CF, %	1.76	2.34	2.98	1.73	2.45	3.05	1.62	2.19	2.86
GE, MJ/kg	16.41	16.49	16.70	16.37	16.47	16.65	316.29	16.39	16.57
Amylose content ^†^, %	68.11	67.94	69.73	49.59	49.15	49.86	33.60	35.89	34.79
Amylopectincontent ^†^, %	31.89	32.06	30.27	50.41	50.85	50.14	66.40	64.11	65.21
Amylose/Amylopectin	2.14	2.12	2.30	0.98	0.97	0.99	0.51	0.56	0.53

NSP: non-starch polysaccharides; MOS: mannan-oligosaccharides; CP: crude protein; NE: net energy; CF: crude fiber; SID: standardized ileal digestible; GE: gross energy. ^‡^ Values for standardized ileal concentrations of amino acids were estimated using standardized ileal digestible coefficients provided by NRC (2012), for amino acids data and feed composition data of NE and CF also obtained from this source. ^†^ The content of amylose and amylopectin were measured using commercial kits of Megazyme (Bray Business Park, Wicklow, Ireland) according to the manufacturer’s instructions.

**Table 4:** Effects of different combinations of starch, NSP, and MOS on the growth performance of weaned pigs.

Items	Different Combinations of Starch, NSP, and MOS									SEM	*p* Values
	T1	T2	T3	T4	T5	T6	T7	T8	T9		
d 0 BW, kg	6.70	6.70	6.71	6.70	6.70	6.70	6.71	6.71	6.71	0.00	0.71
d 28 BW, kg	16.20	17.17	17.24	16.30	16.79	17.44	15.85	16.33	16.09	0.43	0.12
ADFI, g	487.53	533.82	527.41	506.23	527.34	537.27	488.19	489.58	497.73	20.85	0.45
ADG, g	339.29	373.77	376.27	342.94	360.32	383.29	326.43	343.61	335.32	15.57	0.12
G:F	0.695 ^a,b^	0.700 ^a,b^	0.714 ^a^	0.678 ^b^	0.683 ^a,b^	0.713 ^a^	0.668 ^b^	0.702 ^a,b^	0.673 ^b^	0.01	0.02
Diarrhea incidence, %	10.00	9.46	9.05	12.38	11.25	9.28	13.75	11.85	11.01	0.01	0.06

NSP: non-starch polysaccharides; MOS: mannan-oligosaccharides; BW: body weight; ADFI: average daily feed intake; ADG: average daily gain; G:F: gain to feed; SEM: standard error of mean. ^a,b^ Means within a row with different superscripts differ (*p* < 0.05).

**Table 5:** The range analysis of the orthogonal experiment.

**Group**	**Factors and Their Level**			**Results**				
	**A**	**B**	**C**	**d 28 BW (kg)**	**ADFI (g)**	**ADG** **(g)**	**G:F**	**Diarrhea incidence (%)**
T1	A1	B1	C1	16.20	487.53	339.29	0.695	10.00
T2	A1	B2	C2	17.17	533.82	373.77	0.700	9.46
T3	A1	B3	C3	17.24	527.41	376.27	0.714	9.05
T4	A2	B1	C2	16.30	506.23	342.94	0.678	12.38
T5	A2	B2	C3	16.79	527.34	360.32	0.683	11.25
T6	A2	B3	C1	17.44	537.27	383.29	0.713	9.28
T7	A3	B1	C3	15.85	488.19	326.43	0.668	13.75
T8	A3	B2	C1	16.33	489.58	343.61	0.702	11.85
T9	A3	B3	C2	16.09	497.73	335.32	0.673	11.01
**Indicator**	**Value name**		**Factors**					
			**A**		**B**			**C**
d 28 BW (kg)	*k1* ^‡^		16.87		16.12			16.66
	*k2*		16.84		16.76			16.52
	*k3*		16.09		16.92			16.63
	Range *R1* ^†^		0.78		0.81			0.13
	Optimal combination		A1		B3			C1
ADFI (g)	*k1*		516.25		493.98			504.79
	*k2*		523.61		516.91			512.59
	*k3*		491.83		520.80			514.31
	Range *R2*		31.78		26.82			9.52
	Optimal combination		A2		B3			C3
ADG (g)	*k1*		363.11		336.22			355.40
	*k2*		362.18		359.23			350.67
	*k3*		335.12		364.96			354.34
	Range *R3*		27.99		28.74			4.72
	Optimal combination		A1		B3			C1
G:F	*k1*		0.703		0.680			0.703
	*k2*		0.691		0.695			0.684
	*k3*		0.681		0.700			0.688
	Range *R4*		0.0218		0.0198			0.0196
	Optimal combination		A1		B3			C1
Diarrhea incidence (%)	*k1*		9.23		12.04			10.58
	*k2*		11.17		10.85			10.95
	*k3*		12.20		9.70			11.07
	Range *R5*		2.98		2.34			0.50
	Optimal combination		A1		B3			C1

BW: body weight; ADFI: average daily feed intake; ADG: average daily gain; G:F: gain to feed. ^‡^ *k* is the average value of each experimental factor at the same level and used to evaluate the importance of factors. ^†^ *R* is defined as the range between the maximum and minimum values of *k* and a large *R* indicates greater importance of a particular factor. Through comparison of *k* and *R*, the optimal level could be decided for every factor.

**Table 6:** The three-way ANOVA of the orthogonal experiment.

**Group**	**Factors and Their Levels**			**Results**				
	**A**	**B**	**C**	**d 28 BW (kg)**	**ADFI (g)**	**ADG** **(g)**	**G:F**	**Diarrhea Incidence (%)**
**T1**	A1	B1	C1	16.20	487.53	339.29	0.695	10.00
**T2**	A1	B2	C2	17.17	533.82	373.77	0.700	9.46
**T3**	A1	B3	C3	17.24	527.41	376.27	0.714	9.05
**T4**	A2	B1	C2	16.30	506.23	342.94	0.678	12.38
**T5**	A2	B2	C3	16.79	527.34	360.32	0.683	11.25
**T6**	A2	B3	C1	17.44	537.27	383.29	0.713	9.28
**T7**	A3	B1	C3	15.85	488.19	326.43	0.668	13.75
**T8**	A3	B2	C1	16.33	489.58	343.61	0.702	11.85
**T9**	A3	B3	C2	16.09	497.73	335.32	0.673	11.01
**Indicators**		**Sources of variation**		**Three-way ANOVA analysis**				
				**df**	**SS**	**MS**	***F***	***p*-Value**
**d 28 BW (kg)**		A		2	7.02	3.51	2.48	0.09
		B		2	6.57	3.28	2.32	0.11
		C		2	0.17	0.09	0.06	0.94
**ADFI (g)**		A		2	9963.42	4981.71	1.83	0.17
		B		2	7562.62	3781.31	1.39	0.26
		C		2	926.19	463.09	0.17	0.84
**ADG (g)**		A		2	9100.16	4550.08	2.87	0.07
		B		2	8332.28	4166.14	2.62	0.08
		C		2	221.05	110.53	0.07	0.93
**G:F**		A		2	0.0043	0.0022	3.39	0.04
		B		2	0.0039	0.0020	3.09	0.06
		C		2	0.0038	0.0019	3.01	0.06
**Diarrhea incidence (%)**		A		2	65.70	32.85	3.99	0.02
		B		2	46.09	23.04	2.80	0.07
		C		2	8.60	4.30	0.52	0.60

BW: body weight; ADFI: average daily feed intake; ADG: average daily gain; G:F: gain to feed.

**Table 7:** Effects of different combinations of starch, NSP, and MOS on the nutrient apparent digestibility of weaned pigs.

Items	Different Combinations of Starch, NSP, and MOS									SEM	*p* Values
	T1	T2	T3	T4	T5	T6	T7	T8	T9		
DM, %	79.67 ^e^	82.11 ^c,d,e^	80.29 ^e^	85.32 ^a,b,c^	86.38 ^a,b^	88.15^a^	84.63 ^b,c^	83.81 ^b,c,d^	81.10 ^d,e^	0.73	<0.01
EE, %	63.37 ^d^	72.00 ^b,c^	72.37 ^b,c^	70.85 ^b,c^	74.82 ^a,b^	81.16 ^a^	68.47 ^b,c,d^	66.61 ^c,d^	66.27 ^c,d^	1.40	<0.01
Ash, %	45.19 d	50.60 ^c,d^	50.33 ^c,d^	58.42 ^b,c^	61.29 ^a,b^	67.42 ^a^	50.40 ^c,d^	48.17 ^d^	44.91 ^d^	1.87	<0.01
GE, %	78.84 ^e^	81.63 ^c,d,e^	79.58 ^e^	84.31 ^a,b,c^	85.61 ^a,b^	87.79 ^a^	84.03 ^b,c,d^	83.48 ^b,c,d^	80.62 ^d,e^	0.78	<0.01
CP, %	75.07 ^d^	79.06 ^b,c,d^	77.60 ^c,d^	81.89 ^a,b,c^	83.12 ^a,b^	85.98 ^a^	82.17 ^a,b,c^	81.78 ^a,b,c^	78.35 ^b,c,d^	1.05	<0.01

NSP: non-starch polysaccharides; MOS: mannan-oligosaccharides; DM: dry matter; EE: ether extract; Ash: crude ash; GE: gross energy; CP: crude protein; SEM: standard error of mean. ^a–c^ Means within a row with different superscripts differ (*p* < 0.05).

**Table 8:** Effects of different combinations of starch, NSP, and MOS on the blood biochemical parameters of weaned pigs.

Items mmol/L	Different Combinations of Starch, NSP, and MOS									SEM	*p* Values
	T1	T2	T3	T4	T5	T6	T7	T8	T9		
Glu	5.30	5.29	4.93	5.39	5.80	6.01	4.96	5.01	5.94	0.53	0.75
TC	2.49	2.56	2.53	2.23	2.30	2.37	2.41	2.44	2.21	0.14	0.61
TG	0.36	0.31	0.33	0.35	0.36	0.42	0.39	0.38	0.35	0.05	0.90
LDL-c	1.07 ^a^	1.04 ^a^	0.89 ^a,b^	0.79 ^a,b^	0.90 ^a,b^	0.72 ^b^	0.88 ^a,b^	0.90 ^a,b^	0.65 ^b^	0.07	0.01
HDL-c	1.17	1.34	1.52	1.21	1.30	1.33	1.18	1.10	1.12	0.11	0.18

NSP: non-starch polysaccharides; MOS: mannan-oligosaccharides; Glu: glucose; TC: total cholesterol; TG: triglycerides; HDL-c: high density lipoprotein-cholesterol; LDL-c: low density lipoprotein-cholesterol; SEM: standard error of mean. ^a,b^ Means within a row with different superscripts differ (*p* < 0.05).

**Table 9:** Effects of different combinations of starch, NSP, and MOS on the intestinal morphology of weaned pigs.

Items	Different Combinations of Starch, NSP, and MOS									SEM	*p* Values
	T1	T2	T3	T4	T5	T6	T7	T8	T9		
Duodenum											
villus height (um)	232.61	250.11	245.30	207.35	214.58	245.05	216.00	203.31	204.97	13.58	0.08
crypt depth (um)	96.74	97.32	87.76	97.71	94.71	100.78	84.55	88.03	86.69	7.38	0.74
VH:CD	2.46	2.66	2.88	2.17	2.30	2.45	2.59	2.31	2.37	0.17	0.15
Jejunum											
villus height (um)	195.41	224.92	200.20	181.07	217.98	224.47	211.49	201.04	216.31	13.14	0.29
crypt depth (um)	92.03	84.82	90.57	77.42	83.02	93.15	92.19	79.04	81.89	5.07	0.21
VH:CD	2.12	2.68	2.24	2.39	2.65	2.45	2.30	2.56	2.65	0.17	0.19
Ileum											
villus height (um)	162.59	168.34	148.91	153.14	169.21	161.56	183.27	158.56	168.97	9.60	0.37
crypt depth (um)	79.33	72.76	82.22	71.71	74.14	80.12	79.44	76.04	76.30	3.50	0.23
VH:CD	2.07	2.32	1.82	2.15	2.29	2.03	2.33	2.09	2.21	0.13	0.15

NSP: non-starch polysaccharides; MOS: mannan-oligosaccharides; VH: villus height; CD: crypt depth; VH:CD: villus height: crypt depth; SEM: standard error of mean.

**Table 10:** Effects of different combinations of starch, NSP, and MOS on the short-chain fatty acids concentrations of weaned pigs.

Items	Different Combinations of Starch, NSP, and MOS									SEM	*p* Values
	T1	T2	T3	T4	T5	T6	T7	T8	T9		
Serum, umol/L											
Acetate	71.68	63.92	71.44	66.87	63.12	77.07	64.70	65.21	61.90	5.39	0.55
Propionate	94.38	85.21	94.31	88.29	84.82	105.12	84.99	98.84	88.64	7.40	0.52
Butyrate	6.53 ^a,b^	6.67 ^a,b^	5.95 ^a,b^	4.03 ^b^	4.90 ^a,b^	9.84 ^a^	4.06 ^b^	3.30 ^b^	4.36 ^b^	1.16	0.01
Cecum, umol/g											
Acetate	69.96 ^a,b^	79.45 ^a,b^	70.66 ^a,b^	68.31 ^a,b^	69.69 ^a,b^	57.39 ^b^	83.19 ^a^	77.69 ^a,b^	60.75 ^a,b^	4.66	0.02
Propionate	38.66	31.31	44.20	34.07	35.67	29.30	32.09	35.46	29.49	3.33	0.13
Butyrate	22.36	23.97	20.74	23.58	22.99	21.65	15.65	15.62	18.06	1.86	0.33
Colon, umol/g											
Acetate	63.85 ^a,b^	68.09 ^a,b^	61.87 ^b^	70.89 ^a,b^	70.96 ^a,b^	61.84 ^b^	79.74 ^a^	72.98 ^a,b^	67.29 ^a,b^	3.34	0.03
Propionate	32.02	24.62	27.87	26.99	29.97	24.54	26.86	29.68	27.16	1.97	0.29
Butyrate	17.89	16.32	18.48	20.02	18.71	16.16	14.56	14.70	15.93	1.73	0.46

NSP: non-starch polysaccharides; MOS: mannan-oligosaccharides. ^a,b^ Means within a row with different superscripts differ (*p* < 0.05).

## Supplementary Materials

The following are available online at https://www.mdpi.com/2076-2615/10/10/1745/s1, Figure S1: Microbial community bar-plot of top 10 on the class level. The colonic digesta was used for 16S rRNA gene analysis, Figure S2: Microbial community bar-plot of top 10 on the order level. The colonic digesta was used for 16S rRNA gene analysis, Figure S3. Microbial community bar-plot of top 10 on the family level. The colonic digesta was used for 16S rRNA gene analysis, Table 1: Effects of different combinations of starch, NSP and MOS on phyla level (top 10) in microbiota of weaned piglets based on16S rRNA gene, Table S2: Effects of different combinations of starch, NSP and MOS on class level (top 10) in microbiota of weaned piglets based on16S rRNA gene, Table S3: Effects of different combinations of starch, NSP and MOS on order level (top 10) in microbiota of weaned piglets based on16S rRNA gene, Table S4: Effects of different combinations of starch, NSP and MOS on family level (top 10) in microbiota of weaned piglets based on16S rRNA gene, Table S5: Effects of different combinations of starch, NSP and MOS on genus level (top 15) in microbiota of weaned piglets based on16S rRNA gene.
