# Supplementary material for: The Optimal Combination of Dietary Starch, Non-Starch Polysaccharides, and Mannan-Oligosaccharide Increases the Growth Performance and Improves Butyrate-Producing Bacteria of Weaned Pigs
Source: Animals (Basel). 2020 Sep 25;10(10):1745. doi: 10.3390/ani10101745 (PMC7600330; doi:10.3390/ani10101745)
Supplement: Supplementary file 1 [file animals-10-01745-s001.pdf]

Supplementary Material: The Optimal Combination of Dietary Starch, Non-Starch Polysaccharides and Mannan-Oligosaccharide Increases the Growth Performance and Improves Butyrate-Producing Bacteria of Weaned Pigs

Hua Zhou, Bing Yu, Jun He, Xiangbing Mao, Ping Zheng, Jie Yu, Junqiu Luo, Yuheng Luo, Hui Yan and Daiwen Chen

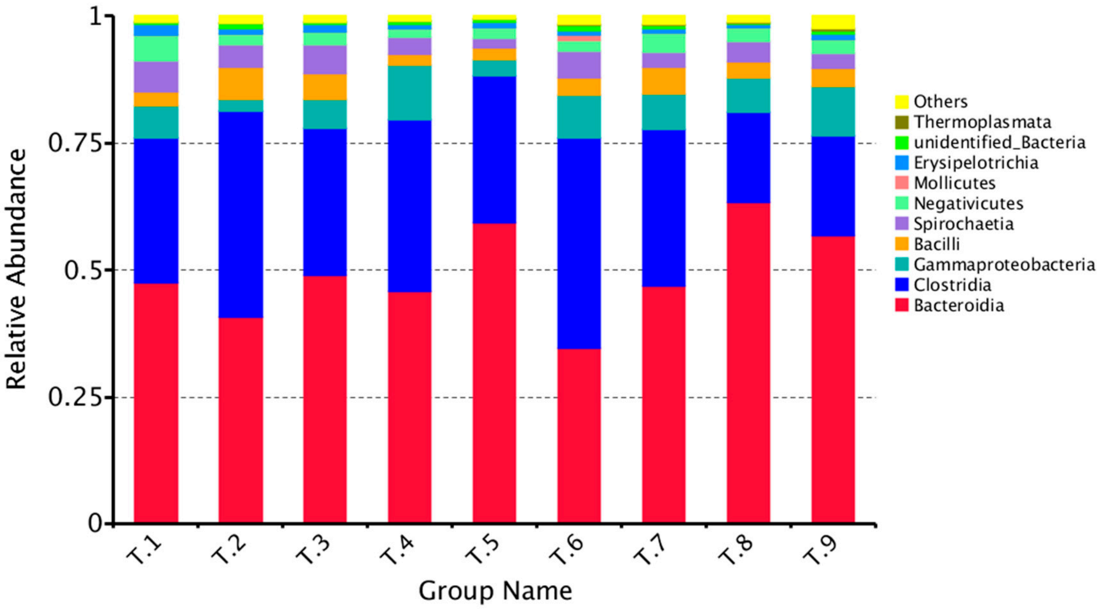

Figure S1. Microbial community bar-plot of top 10 on the class level. The colonic digesta was used for 16S rRNA gene analysis.

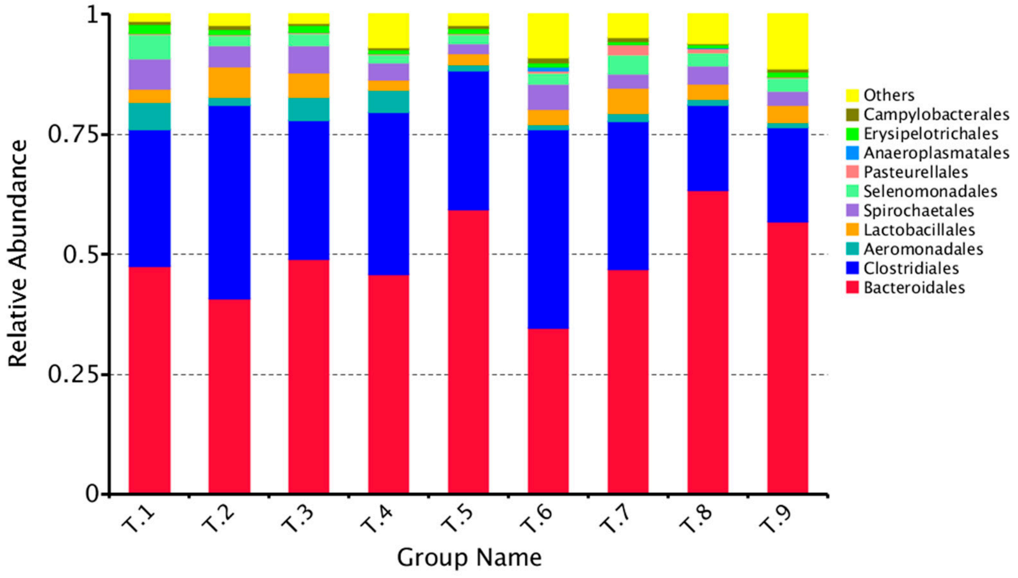

Figure S2. Microbial community bar-plot of top 10 on the order level. The colonic digesta was used for 16S rRNA gene analysis.

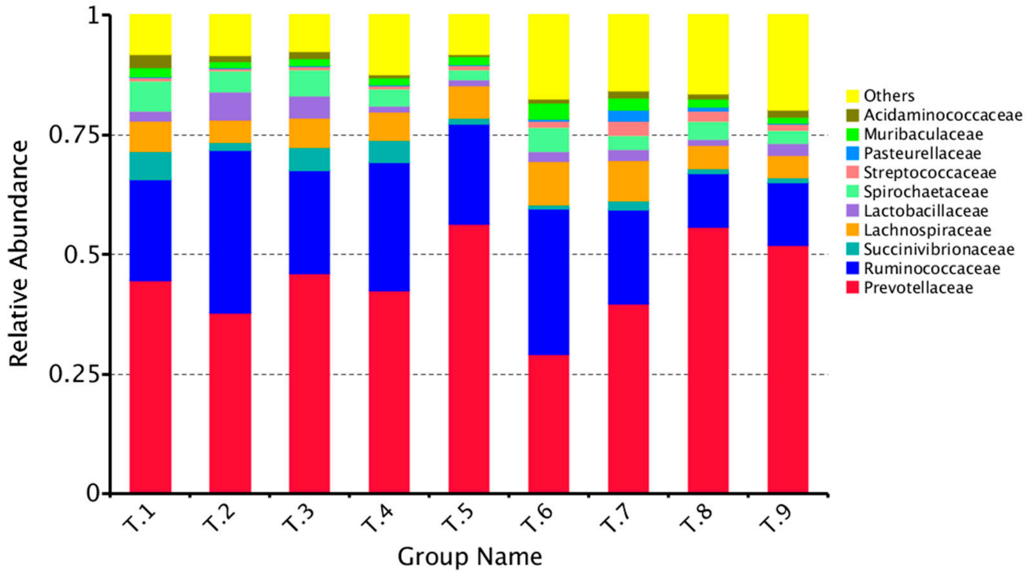

Figure S3. Microbial community bar-plot of top 10 on the family level. The colonic digesta was used for 16S rRNA gene analysis.

Table 1. Effects of different combinations of starch, NSP and MOS on phyla level (top 10) in microbiota of weaned piglets based on16S rRNA gene.

Items	Different combinations of starch, NSP and MOS									SEM	*p* Values
	T1	T2	T3	T4	T5	T6	T7	T8	T9		
Bacteroidetes	0.4758 [a,b,c,d]	0.4079 [c,d]	0.4910 [a,b,c,d]	0.4578 [b,c,d]	0.5935 [a,b]	0.3473 [d]	0.4689 [a,b,c,d]	0.6334 [a]	0.5682 [a,b,c]	0.0376	<0.01
Firmicutes	0.3825 [a,b,c]	0.4777 [a]	0.3796 [a,b,c]	0.3846 [a,b,c]	0.3443 [a,b,c]	0.4791 [a]	0.4062 [a,b]	0.2446 [c]	0.2710 [b,c]	0.0346	<0.01
Proteobacteria	0.0681	0.0350	0.0633	0.1129	0.0368	0.0969	0.0807	0.0723	0.1076	0.0264	0.37
Spirochaetes	0.0631	0.0455	0.0556	0.0352	0.0200	0.0522	0.0300	0.0388	0.0278	0.0115	0.17
Tenericutes	0.0004	0.0002	0.0006	0.0003	0.0006	0.0091	0.0007	0.0005	0.0004	0.0029	0.41
Cyanobacteria	0.0075	0.0101	0.0073	0.0039	0.0027	0.0120	0.0043	0.0035	0.0149	0.0034	0.16
Euryarchaeota	0.0004	0.0009	0.0006	0.0009	0.0006	0.0012	0.0034	0.0020	0.0051	0.0013	0.19
Fibrobacteres	0.0003	0.0009	0.0009	0.0032	0.0006	0.0008	0.0028	0.0012	0.0025	0.0010	0.33
Fusobacteria	0.0000 [b]	0.0000 [b]	0.0000 [b]	0.0000 [b]	0.0000 [b]	0.0000 [b]	0.0007 [b]	0.0024 [a]	0.0009 [b]	0.0002 [b]	<0.01
Actinobacteria	0.0010	0.0007	0.0006	0.0004	0.0006	0.0005	0.0005	0.0003	0.0005	0.0002	0.26

NSP: non-starch polysaccharide; MOS: mannan-oligosaccharides. [a–d] Means within a row with different superscripts differ ($p < 0.05$).

Table S2. Effects of different combinations of starch, NSP and MOS on class level (top 10) in microbiota of weaned piglets based on16S rRNA gene.

Items	Different combinations of starch, NSP and MOS									SEM	*p* Values
	T1	T2	T3	T4	T5	T6	T7	T8	T9		
Bacteroidia	0.4758 [a,b,c,d]	0.4079 [c,d]	0.4909 [a,b,c,d]	0.4578 [b,c,d]	0.5934 [a,b]	0.3472 [d]	0.4688 [a,b,c,d]	0.6334 [a]	0.5682 [a,b,c]	0.0376	<0.01
Clostridia	0.2844 [a,b,c]	0.4045 [a]	0.2875 [a,b,c]	0.3389 [a,b]	0.2895 [a,b,c]	0.4137 [a]	0.3084 [a,b,c]	0.1775 [c]	0.1952 [b,c]	0.0343	<0.01
Gammaproteobacteria	0.0621	0.0235	0.0558	0.1054	0.0298	0.0821	0.0654	0.0642	0.0918	0.0262	0.4254
Bacilli	0.0274	0.0622	0.0522	0.0208	0.0232	0.0332	0.0520	0.0324	0.0375	0.0113	0.1492
Unidentified Spirochaetes	0.0631	0.0455	0.0556	0.0352	0.0201	0.0522	0.0300	0.0388	0.0278	0.0115	0.1769
Negativicutes	0.0501 [a]	0.0212 [b,c]	0.0259 [b,c]	0.0167 [c]	0.0207 [b,c]	0.0228 [b,c]	0.0389 [a,b]	0.0282 [b,c]	0.0276 [b,c]	0.0044	<0.01

Mollicutes	0.0004	0.0002	0.0006	0.0003	0.0001	0.0091	0.0007	0.0005	0.0004	0.0028	0.4141
Melainabacteria	0.0075	0.0101	0.0073	0.0039	0.0027	0.0120	0.0043	0.0035	0.0149	0.0034	0.1652
Erysipelotrichia	0.0207 [a]	0.0098 [a,b]	0.0141 [a,b]	0.0081 [b]	0.0109 [a,b]	0.0095 [a,b]	0.0068 [b]	0.0065 [b]	0.0107 [a,b]	0.0025	<0.01
Epsilonproteobacteria	0.0045	0.0097	0.0049	0.0050	0.0047	0.0104	0.0068	0.0028	0.0065	0.0019	0.1229

NSP: non-starch polysaccharide; MOS: mannan-oligosaccharides. [a–d] Means within a row with different superscripts differ ($p < 0.05$).

Table S3. Effects of different combinations of starch, NSP and MOS on order level (top 10) in microbiota of weaned piglets based on16S rRNA gene.

Items	Different combinations of starch, NSP and MOS									SEM	*p* Values
	T1	T2	T3	T4	T5	T6	T7	T8	T9		
Bacteroidales	0.4755 [a,b,c]	0.4065 [b,c]	0.4902 [a,b,c]	0.4564 [a,b,c]	0.5929 [a]	0.3449 c	0.4663 [a,b,c]	0.6292 [a]	0.5583 [a,b]	0.0381	<0.01
Clostridiales	0.2843 [a,b,c]	0.4045 [a]	0.2875 [a,b,c]	0.3389 [a,b]	0.2895 [a,b,c]	0.4136 [a]	0.3083 [a,b,c]	0.1775 [c]	0.1951 [b,c]	0.0343	<0.01
Aeromonadales	0.0605	0.0220	0.0532	0.1025	0.0277	0.0779	0.0418	0.0548	0.0885	0.0262	0.4108
Lactobacillales	0.0274	0.0622	0.0522	0.0208	0.0232	0.0332	0.0520	0.0321	0.0370	0.0113	0.1488
Spirochaetales	0.0631	0.0455	0.0556	0.0352	0.0201	0.0522	0.0300	0.0388	0.0278	0.0115	0.1769
Selenomonadales	0.0501 [a]	0.0212 [b,c]	0.0259 [b,c]	0.0167 [c]	0.0207 [b,c]	0.0228 [b,c]	0.0389 [a,b]	0.0282 [b,c]	0.0276 [b,c]	0.0044	<0.01
Pasteurellales	0.0013	0.0011	0.0022	0.0023	0.0018	0.0035	0.0220	0.0084	0.0026	0.0047	0.0617
Anaeroplasmatales	0.0001	0.0000	0.0003	0.0002	0.0000	0.0088	0.0004	0.0001	0.0001	0.0028	0.4220
Gastranaerophilales	0.0075	0.0101	0.0073	0.0039	0.0027	0.0120	0.0043	0.0035	0.0149	0.0034	0.1652
Erysipelotrichales	0.0207 [a]	0.0098 [a,b]	0.0141 [a,b]	0.0081 [b]	0.0109 [a,b]	0.0095 [a,b]	0.0068 [b]	0.0065 [b]	0.0107 [a,b]	0.0025	<0.01

NSP: non-starch polysaccharide; MOS: mannan-oligosaccharides. [a–c] Means within a row with different superscripts differ ($p < 0.05$).

Table S4. Effects of different combinations of starch, NSP and MOS on family level (top 10) in microbiota of weaned piglets based on16S rRNA gene.

Items	Different combinations of starch, NSP and MOS									SEM	*p* Values
	T1	T2	T3	T4	T5	T6	T7	T8	T9		
Prevotellaceae	0.4450 [a,b]	0.3761 [a,b]	0.4590 [a,b]	0.4248 [a,b]	0.5617 [a]	0.2902 [b]	0.3975 [a,b]	0.5570 [a]	0.5190 [a]	0.0419	<0.01
Ruminococcaceae	0.2101 [a,b,c]	0.3422 [a]	0.2146 [a,b,c]	0.2663 [a,b]	0.2092 [a,b,c]	0.3043 [a]	0.1960 [a,b,c]	0.1110 [c]	0.1305 [b,c]	0.0326	<0.01

Succinivibrionaceae	0.0605	0.0220	0.0531	0.1025	0.0277	0.0779	0.0418	0.0546	0.0885	0.0262	0.4108
Lachnospiraceae	0.0645	0.0460	0.0612	0.0585	0.0675	0.0899	0.0855	0.0480	0.0463	0.0096	0.1448
Lactobacillaceae	0.0220 ^{a,b}	0.0578 ^{a}	0.0469 ^{a,b}	0.0130 ^{b}	0.0139 ^{b}	0.0206 ^{a,b}	0.0227 ^{a,b}	0.0126 ^{b}	0.0253 ^{a,b}	0.0094	0.0134
Spirochaetaceae	0.0631	0.0455	0.0556	0.0352	0.0201	0.0522	0.0300	0.0388	0.0278	0.0115	0.1769
Streptococcaceae	0.0054	0.0044	0.0053	0.0079	0.0093	0.0126	0.0293	0.0194	0.0116	0.0061	0.1117
Pasteurellaceae	0.0013	0.0011	0.0022	0.0023	0.0018	0.0035	0.0220	0.0084	0.0026	0.0047	0.0617
Bacteroidales _S24-7_group	0.0203	0.0136	0.0142	0.0149	0.0159	0.0332	0.0262	0.0262	0.0119	0.0053	0.0857
Acidaminococcaceae	0.0265 ^{a}	0.0130 ^{a,b}	0.0147 ^{a,b}	0.0055 ^{b}	0.0056 ^{b}	0.0100 ^{b}	0.0150 ^{a,b}	0.0118 ^{a,b}	0.0150 ^{a,b}	0.0032	<0.01

NSP: non-starch polysaccharide; MOS: mannan-oligosaccharides. $^{a–c}$ Means within a row with different superscripts differ (p < 0.05).

Table S5. Effects of different combinations of starch, NSP and MOS on genus level (top 15) in microbiota of weaned piglets based on16S rRNA gene.

**Items**	**Different combinations of starch, NSP and MOS**									**SEM**	***p* Values**
	**T1**	**T2**	**T3**	**T4**	**T5**	**T6**	**T7**	**T8**	**T9**		
Prevotella_9	0.3197 ^{a,b}	0.2627 ^{a,b}	0.3015 ^{a,b}	0.2786 ^{a,b}	0.4073 ^{a}	0.1748 ^{b}	0.1736 ^{b}	0.2322 ^{b}	0.2793 ^{a,b}	0.0342	<0.01
Ruminococcus_2	0.1019 ^{b,c}	0.2656 ^{a}	0.1078 ^{b,c}	0.1966 ^{a,b}	0.1345 ^{a,b,c}	0.1923 ^{a,b}	0.0965 ^{b,c}	0.0478 ^{c}	0.0602 ^{b,c}	0.0309	<0.01
Succinivibrionaceae _UCG-002	0.0019	0.0056	0.0042	0.0554	0.0149	0.0685	0.0238	0.0427	0.0773	0.0204	0.07
Alloprevotella	0.0101 ^{b}	0.0103 ^{b}	0.0139 ^{b}	0.0228 ^{b}	0.0165 ^{b}	0.0214 ^{b}	0.0405 ^{a,b}	0.0677 ^{a}	0.0382 ^{a,b}	0.0084	<0.01
Succinivibrio	0.0585	0.0164	0.0488	0.0469	0.0127	0.0092	0.0179	0.0118	0.0111	0.0145	0.1
Prevotellaceae_NK3 B31_group	0.0101 ^{b}	0.0097 ^{b}	0.0106 ^{b}	0.0174 ^{b}	0.0227 ^{b}	0.0120 ^{b}	0.0366 ^{b}	0.0817 ^{a}	0.0261 ^{b}	0.0079	<0.01
Prevotella_2	0.0229 ^{c}	0.0229 ^{c}	0.0316 ^{b,c}	0.0339 ^{b,c}	0.0317 ^{b,c}	0.0344 ^{b,c}	0.0560 ^{a,b}	0.0525 ^{b,c}	0.0879 ^{a}	0.0071	<0.01
Lactobacillus	0.0220 ^{a,b}	0.0578 ^{a}	0.0469 ^{a,b}	0.0130 ^{b}	0.0139 ^{b}	0.0206 ^{a,b}	0.0227 ^{a,b}	0.0126 ^{b}	0.0253 ^{a,b}	0.0094	<0.01
Treponema_2	0.0621	0.0439	0.0507	0.0336	0.0175	0.0494	0.0239	0.0244	0.0235	0.0115	0.1

Prevotella_7	0.0189	0.0157	0.0222	0.0196	0.0313	0.0132	0.0222	0.0415	0.0288	0.0073	0.2
Streptococcus	0.0054	0.0044	0.0053	0.0079	0.0093	0.0126	0.0293	0.01940	0.0116	0.0061	0.11
Actinobacillus	0.0013	0.0011	0.0022	0.0022	0.0017	0.0035	0.0219	0.0082	0.0026	0.0047	0.06
Prevotella_1	0.0032 [b]	0.0042 [b]	0.0036 [b]	0.0083 [b]	0.0063 [b]	0.0058 [b]	0.0276 [a]	0.0259 [a]	0.0151 [a,b]	0.0030	<0.01
Succiniclasticum	0.0188 [a]	0.0089 [a,b]	0.0111 [a,b]	0.0016 [b]	0.0015 [b]	0.0034 [b]	0.0008 [b]	0.0000 [b]	0.0038 [b]	0.0031	<0.01
Anaeroplasma	0.0001	0.0000	0.0003	0.0002	0.0000	0.0088	0.0004	0.0001	0.0001	0.0028	0.42

NSP: non-starch polysaccharide; MOS: mannan-oligosaccharides. [a–c] Means within a row with different superscripts differ ($p < 0.05$).

© 2020 by the authors. Licensee MDPI, Basel, Switzerland. This article is an open access article distributed under the terms and conditions of the Creative Commons Attribution (CC BY) license (http://creativecommons.org/licenses/by/4.0/).
